# Which environmental factors control extreme thermal events in rivers? A multi-scale approach (Wallonia, Belgium)

**DOI:** 10.7717/peerj.12494

**Published:** 2021-11-22

**Authors:** Blandine Georges, Adrien Michez, Hervé Piegay, Leo Huylenbroeck, Philippe Lejeune, Yves Brostaux

**Affiliations:** 1 University of Liège (ULiege), Gembloux Agro-Bio Tech, TERRA Teaching and Research Centre, Gembloux, Belgium; 2 Université Rennes II-Haute-Bretagne, Rennes, France; 3 University of Lyon, Ecole Normale Supérieure de Lyon, Lyon, France

**Keywords:** Stream water temperature, Riparian vegetation, Channel morphology, Shade, Extreme, Environmental factors, River management

## Abstract

Managers need to know how to mitigate rising stream water temperature (WT) due to climate change. This requires identifying the environmental drivers that influence thermal regime and determining the spatial area where interventions are most effective. We hypothesized that (i) extreme thermal events can be influenced by a set of environmental factors that reduce thermal sensitivity and (ii) the role played by those factors varies spatially. To test these hypotheses, we (i) determined which of the environmental variables reported to be the most influential affected WT and (ii)identified the spatial scales over which those environmental variables influenced WT. To this end, the influence of multi-scale environmental variables, namely land cover, topography (channel slope, elevation), hydromorphology (channel sinuosity, water level, watershed area, baseflow index) and shade conditions, was analyzed on the three model variables (day thermal sensitivity, night thermal sensitivity, and non-convective thermal flux) in the model developed by Georges et al. (2021) of the temporal thermal dynamics of daily maximum WT during extreme events. Values were calculated on six spatial scales (the entire upstream catchment and the associated 1 km and 2 km circular buffer, and 50 m wide corridors on each side of the stream with the associated 1 km and 2 km circular buffer). The period considered was 17 extreme days during the summer identified by Georges et al. (2021) based on WT data measured every 10 min for 7 years (2012–2018) at 92 measurement sites. Sites were located evenly throughout the Wallonia (southern Belgium) hydrological network. Results showed that shade, baseflow index (a proxy of the influence of groundwater), water level and watershed area were the most significant variables influencing thermal sensitivity. Since managers with finite financial and human resources can act on only a few environmental variables, we advocate restoring and preserving the vegetation cover that limits solar radiation on the watercourse as a cost-effective solution to reduce thermal sensitivity. Moreover, management at small spatial scale (50 m riparian buffer) should be strategically promoted (for finance and staffing) as our results show that a larger management scale is not more effective in reducing thermal sensitivity to extreme events.

## Introduction

Atmospheric heating caused by climate changes elevates stream water temperature (WT) (Mohseni, Stefan & Eaton, 2003). Van Vliet et al. (2011) predicted increases of 1.3 °C, 2.6 °C and 3.8 °C in mean annual river temperatures under respectively 2 °C, 4 °C and 6 °C increases in air temperature. In climate projections of a doubled atmospheric CO_2_ level, some rivers in Minnesota (USA) could heat up by 2.4 °C to 4.7 °C (mean summer WT) (Stefan & Sinokrot, 1993). Numerous other studies point to a significant increase in WT in the coming years (Morrison, Quick & Foreman, 2002; Mohseni, Stefan & Eaton, 2003; Mantua, Tohver & Hamlet, 2010; Isaak et al., 2012). However, in the United States (Kaushal et al., 2010) and Europe (Webb, 1996) WT increases are already being recorded.

WT increase can damage the aquatic ecosystem especially for ectothermic species (Isaak et al., 2012). WT is an essential variable that regulates the breeding, metabolism, feeding, migration and survival of fish species (Jonsson, 1991; Gillooly et al., 2001; Wolter, 2007; Luo et al., 2013). WT also influences the behavior of aquatic insects (Dallas, 2008), invertebrates (Brockington & Clarke, 2001), and the presence of blue-green algae (McQueen & Lean, 1987).

Besides warming, climate change is expected to cause an increased frequency of extreme events (Weaver, Kumar & Chen, 2014; Zhou et al., 2020), defined as events outside the normal or seasonal range (Georges et al., 2021). Zhou et al. (2020) reviews several studies showing that global mean change has less impact on society and ecosystems than extreme weather events. Very few studies have analyzed such extreme events in aquatic ecosystems (Caissie, Ashkar & El-Jabi, 2020; Georges et al., 2021). They can occur at any season, and so are important for ectothermal fish, whose life cycle is attuned to seasonal patterns (Kramer, 1978; Conover, 1992). Winemiller (1989) even refers to “seasonal strategy” fish. For example, Davies & Bromage (2002) show a dysfunction in ovarian development that can leave no viable eggs if spawning time is advanced because the temperatures are optimal for this development phase but are above the seasonal norms. The strong impact of seasonality on aquatic ecosystems makes analysis of extreme events particularly important.

River warming and extreme events are key challenges. Managers need to know how to mitigate rising stream WT to preserve their aquatic ecosystems (Boon & Raven, 2012). The main current management principle is to try to reduce warming by increasing shading, raising minimum flow, and reducing hot spots by working in particular on riparian vegetation (Orr et al., 2015). However, scientific knowledge is constantly advancing, and climate change is causing aquatic ecosystems to continuously change (Boon & Raven, 2012). The actions put in place by managers may therefore need to evolve and be tested to assess their effectiveness in the light of ongoing climate change (Orr et al., 2015).

In this context, research needs to identify the environmental drivers that influence thermal regime and determine the spatial area where interventions will be most effective.

Determining the environmental drivers of extreme WT is generally considered a key challenge for river managers seeking effective management measures to mitigate WT rise during warm events. Different drivers of WT have already been studied in earlier research. Because energy exchanges occur over the air-water interface mainly due to solar radiation, **air temperature** is a useful surrogate to predict WT fluctuations (LeBlanc, Brown & FitzGibbon, 1997; Erickson & Stefan, 2000; Johnson, 2003). Air temperature is classically used to model WT (Caissie, Satish & El-Jabi, 2007). For example, Webb et al. (2008) reviewed different studies modeling the air-water relation with linear regression. Mayer (2012) pointed out in their study that the air-water temperature relationship is different at regional or individual stream scales and also according to whether air temperature alone is being considered or whether other variables are incorporated to study their influence on WT. For example, air temperature influences WT more markedly when flow rate is low. Slowing **flow** generally increases river heating because it decreases water depth (Cristea & Janisch, 2007).

**Elevation** also shows a significant correlation with stream temperature as demonstrated by Donato (2002) and O’Sullivan, Devito & Curry (2019). Higher elevation monitoring stations tend to record lower air temperatures. WT is therefore generally lower (Siegel & Volk, 2019) when elevation is higher. Stream **slope**, another topographic characteristic of watercourses, also influences WT (Mayer, 2012). The correlation between the two variables is negative. When the slope is steep, the water moves faster and has a shorter residence time to absorb heat from its environment (Donato, 2002; Pratt & Chang, 2012). Slope and elevation are correlated variables as reported by Alber & Piégay (2011), who have developed a method for delineating and characterizing fluvial features at a regional scale (over 1,000 km^2^) using raw data.

Several studies have also investigated the role of **land cover** on WT. LeBlanc, Brown & FitzGibbon (1997) modeled the impact of land use on temperature in urban streams during extreme events. Li et al. (2008) showed that WT was significantly related to plant cover. Other studies underscore the role of riparian vegetation in limiting extreme WT in streams (Arora et al., 2018; Brazier & Brown, 1973; Bartholow, 2000; Garner et al., 2017; Bond, Stubblefield & Van Kirk, 2015; LeBlanc, Brown & FitzGibbon, 1997; Moore, Spittlehouse & Story, 2005; Malcolm et al., 2008). Riparian vegetation lowers wind speed, raises relative humidity and brings shade through radiation interception by foliage, which also prevents thermal heating of rivers (Brosofske et al., 1997). Some research shows that shade strongly influences stream WT (Brown & Krygier, 1970; Beschta, 1997; Poole & Berman, 2001) by influencing solar energy inputs into the water (Brazier & Brown, 1973; Brosofske et al., 1997). It is observed that shaded streams tend to be cooler in summer (Siegel & Volk, 2019). Of all those enumerated above, land cover is one of the few variables on which humans can act to mitigate high WT without altering the properties of the stream itself.

The **hyporheic zone** also controls a river’s thermal dynamics. This zone is the locus of complex exchanges (water, nutrients, organic matter, etc.) between groundwater (channel bed or banks) and surface water (Boulton et al., 1998; Burkholder et al., 2008). Hyporheic exchange influences spatial and temporal WT variability (Cristea & Janisch, 2007). Especially during the summer months, cooler groundwater mixed with warmer mainstream water dampens WT rise and decreases maximum WT (Johnson, 2004). Numerous studies demonstrate that the hyporheic zone plays an important role in mitigating WT rise (Story, Moore & Macdonald, 2003; Hester, Doyle & Poole, 2009; White, Elzinga & Hendricks, 1987). Some recent studies use thermal infrared sensors to detect thermal heterogeneities due to hyporheic exchanges (Marteau et al., 2021; Dole-Olivier et al., 2019; Eschbach et al., 2017). In addition, different studies have shown that **sinuosity**, an important hydromorphologic component of rivers (Aswathy, Vijith & Satheesh, 2008), is an essential driver of the exchanges in the hyporheic zone (Boano et al., 2006; Kasahara & Wondzell, 2003). Sinuosity also influences WT by increasing total channel length and thereby enabling exposure to solar radiation for a longer period of time (residence time) (Wondzell, Diabat & Haggerty, 2019).

**Discharge** also influences WT, because high discharge leads to higher inertia to air temperature (Sinokrot & Stefan, 1994). WT change is inversely proportional to streamflow (LeBlanc, Brown & FitzGibbon, 1997). Gu, Montgomery & Austin (1998) demonstrated that the variation of daily maximum WT with discharge was stronger than that of daily mean WT.

Although many studies have analyzed the environmental variables that influence river WT studied one by one, only some of them consider stream WT as a function of a set of variables fully representative of the environment. Knowledge of the mutual influence of multiple variables on WT is important for effective water management measures. For example, Poole & Berman (2001), Donato (2002), and Sinokrot & Stefan (1994) found a correlation between WT and several environmental variables such as slope, drainage area, air temperature, discharge and solar radiation. Garner et al. (2017) studied the influence of riparian vegetation density, channel orientation and flow velocity on river temperature. Risley, Roehl & Conrads (2003) estimated WT through artificial neural networks with stream habitat and basin variables. However, from previous studies, it is not clear which environmental variable has the greatest influence on WT, and there are numerous conflicting findings (Johnson, 2003), in particular because these variables vary greatly in time and space, yielding different results between study sites.

WT is influenced by environmental variables over a range of spatial scales. Some environmental factors may influence the stream at a large scale, while others act locally (Kovach et al., 2018; Sliva & Williams, 2001). Furthermore, interactions occur between large-scale and local factors (Frissell et al., 1986). In their study on the impact of land use on river water quality (including WT) comparing buffer zone and whole catchment, Sliva & Williams (2001) demonstrated that the 100 m buffer landscape characteristics had slightly less influence on water quality than the catchment scale. Kovach et al. (2018) analyzed the effects of roads on summer thermal regime by quantifying them on three spatial scales (stream segment, riparian buffer, and catchment). Chang & Psaris (2013) focused on four spatial scales (relative contributing area (RCA) scale, RCA buffered scale, 1 km upstream RCA scale and 1 km upstream buffer scale) to understand local landscape predictors of maximum stream temperature. However, the issue is controversial since the spatial scale at which each environmental factor operates is still unclear (Chang & Psaris, 2013; Sliva & Williams, 2001) because the relationship between variables and spatial scales is complex. For example, Isaak et al. (2010) show that local conditions (1 km immediately upstream) influenced WT more in the Boise River basin in central Idaho (USA), while Scott et al. (2002) report the opposite (stream physics and chemistry including WT were better explained by predictors at the catchment scale than by more localized ones) in the Upper Tennessee in western North Carolina (USA).

The influence of a wide range of environmental variables on the evolution of WT during extreme events still needs to be explored quantitatively in a multi-scale perspective to gain a better understanding of the vulnerability or resilience of rivers to warming around extreme events. To this end, we explored the influence of multi-scale environmental variables on the dynamics of WT extreme events, through the study of the three model variables in the model of Georges et al. (2021), namely day thermal sensitivity, night thermal sensitivity, and non-convective thermal flux. They carried out a correlation analysis (i) between the model variables and the number of extreme days, and (ii) between the model variables and the number of days above a fixed thermal threshold to determine whether the variables adequately reflected sensitivity to stream heating. Georges et al. (2021) demonstrated that generalized additive models were particular useful for identifying extreme WT in rivers. They also developed a model to analyze the temporal thermal dynamics of day maximum WT during extreme events. The model, based on differential equations, has the advantage of taking WT temporal dynamics into account and is simple to apply because it uses commonly recorded variables: air temperature and streamflow. This model was applied to WT data recorded between 2012 and 2018 in Wallonia (Southern Belgium) on a network of 92 measurement sites, giving estimates for variables related to the thermal sensitivity of each site. However, the three model variables estimated (day thermal sensitivity, night thermal sensitivity, and non-convective thermal flux) were not studied in relation to environmental characteristics to identify and rank their components.

From the previous studies cited above, we hypothesized that extreme thermal events were linked to air temperature and could be smoothed by a set of environmental variables such as shade and groundwater inflows, which reduce thermal sensitivity. We also hypothesized that the role played by those factors varied spatially.

To test these hypotheses, we specifically set out to:

1. Determine which environmental variables among the most influential ones reported in the literature affected WT dynamics around extreme events;
2. Identify the spatial scales over which those environmental variables influenced WT dynamics around extreme events.

Our purpose was to help river managers gain a better understanding of the environmental variables and the scale of action needed to develop decision-making tools needed to preserve rivers from the effects of future climate change.

## Materials & Methods

### Study area

**Figure 1:**
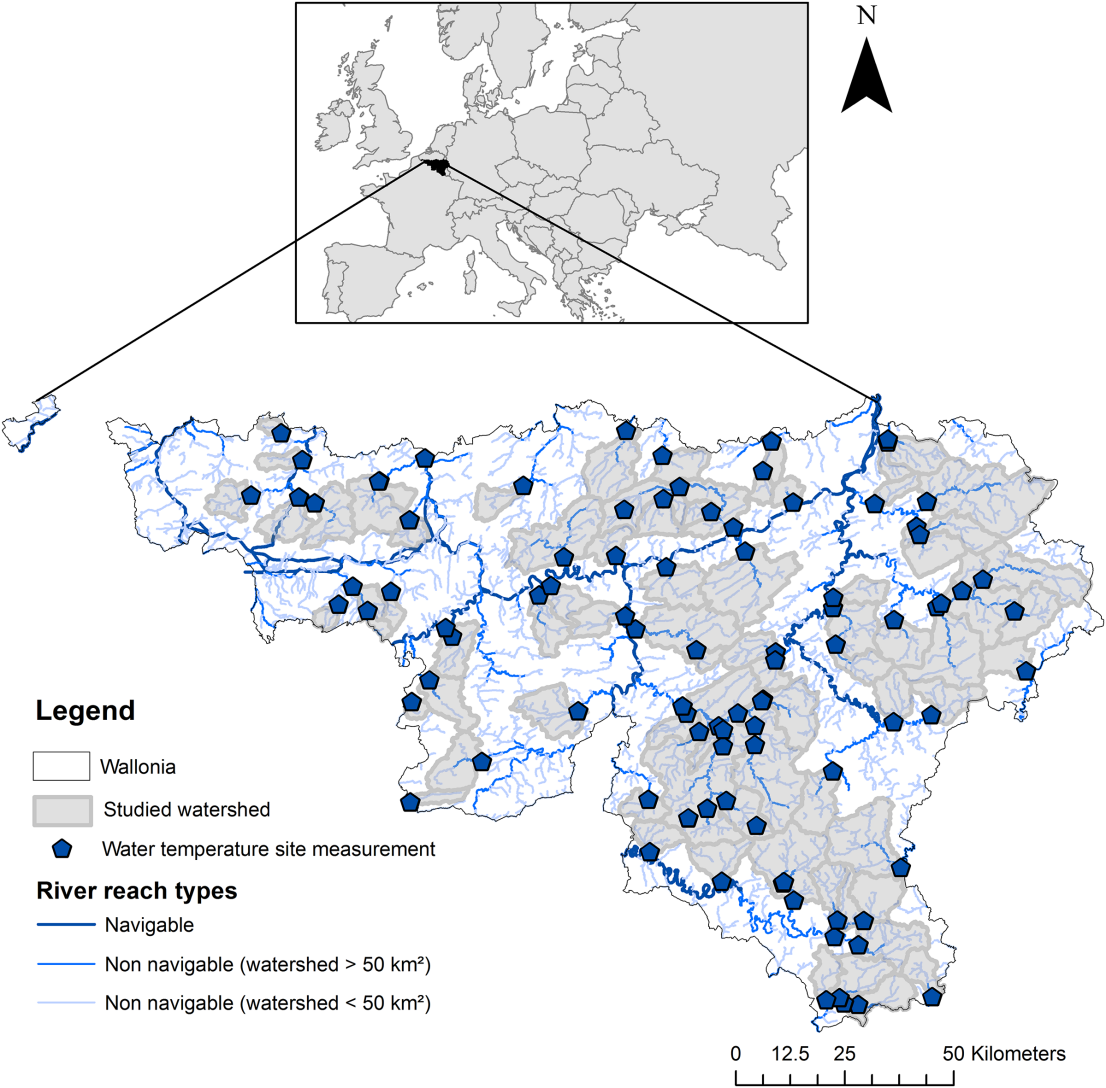
Location of the WT site measurements and their watersheds in Wallonia (Southern Belgium). Source credit: Georges et al. (2021), copyright Elsevier.

**Figure 2:**
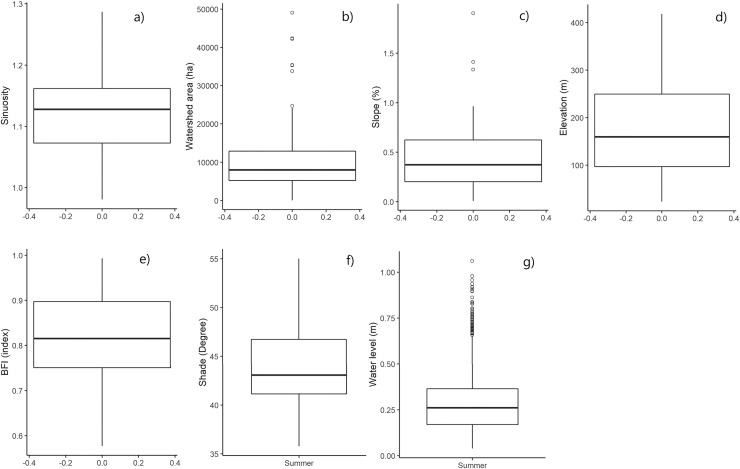
Boxplots showing the variability of the sinuosity (A), watershed area (B), slope (C), elevation (D), baseflow index (BFI) (E), shade (F) and water level (G). For the figure, shade and sinuosity were calculated only for the *CA* spatial scale for the 92 measurement sites. “Summer” was mentioned for parameters varying temporally.

**Figure 3:**
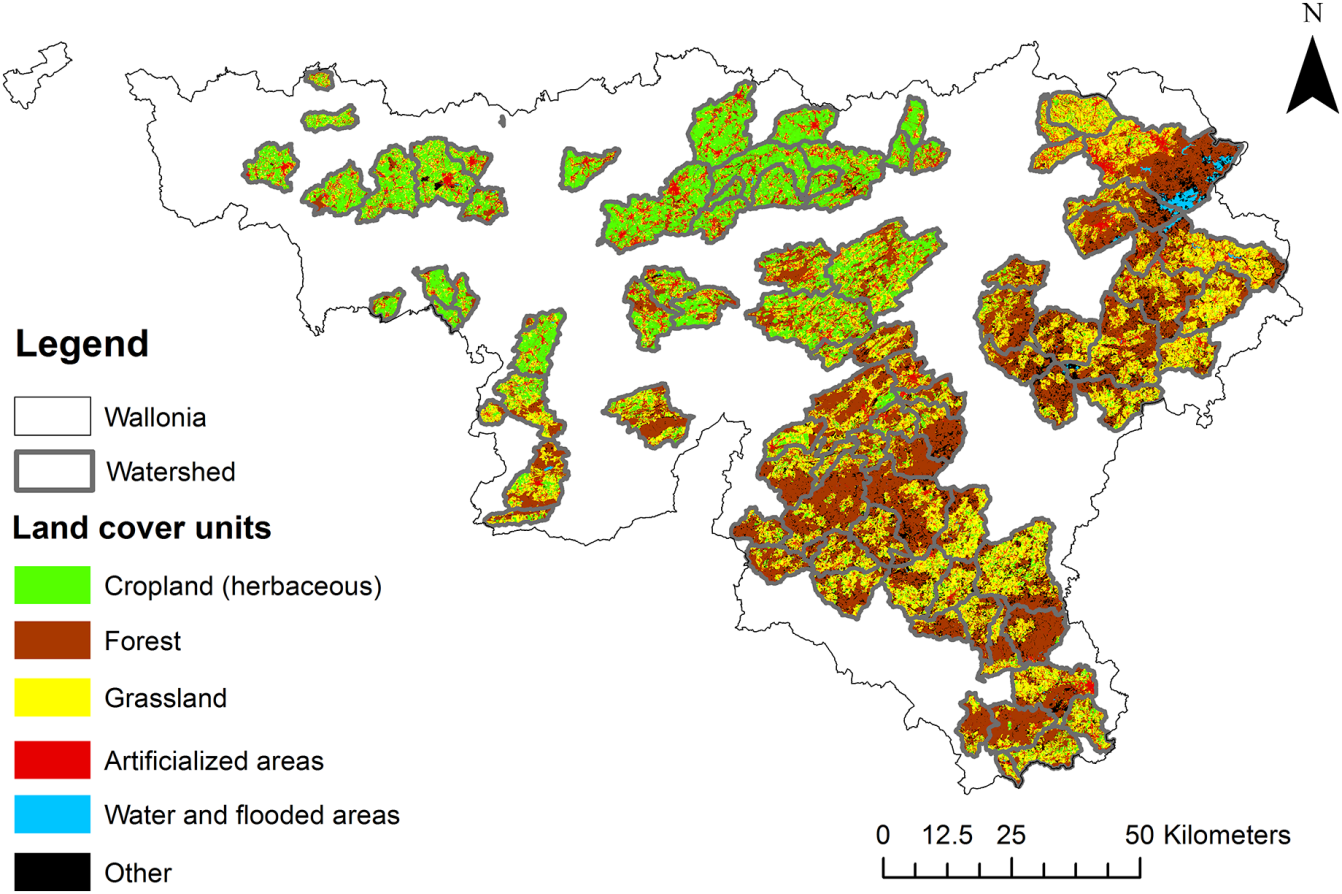
Land cover classes for the 92 watersheds studied (Lifewatch project; version 2.10, 2015; Delangre, Radoux & Dufrêne, 2017; Radoux & Bogaert, 2017). Cropland: herbaceous; mixed herbaceous and tree cover (majority of herbaceous). Forest: broadleaved deciduous forest; needleleaved sempervirens forest; mixed forest; mixed herbaceous and tree cover (majority of trees). Grassland: mixed crop cover; permanent monospecific productive grassland. Artificialized areas. Water and flooded areas: shrub and herbaceous flooded; water. Other: recently cleared areas with forest regrowth, shrubs, Christmas trees, mixture of vegetation and bare soils.

The study focused on Wallonia, the southern region of Belgium (Europe), which covers an area of 16,901 km². It was based on 92 measurement sites, each of which delimited a watershed (Fig. 1). The watersheds had a total drainage area of 11,309 km². Elevation and topographic slope varied strongly among the studied measurement sites (Fig. 2), as well as land use and shading conditions (Fig. 3). The studied watersheds ranged widely in their size (surface area) and their physical conditions (channel sinuosity, flow regime and water level) (Fig. 2).

### Model studied/temporal thermal dynamic characterization

The spatiotemporal ranges of the variables associated with an empirical model (Eq. (1)) developed by Georges et al. (2021) were studied to understand the temporal thermal dynamics over the study area. This model describes dynamics of WT around extreme climatic (temperature) events. The extreme events were identified with generalized additive models (GAMs). Further details on the temporal thermal dynamic model and the identification of extreme events are described in Georges et al. (2021).

(1) $${\displaystyle{{d{T_w}} \over {dt}} = \displaystyle{{\left[ {a\left( {{T_{aM}}-{T_w}} \right) + b\left( {{T_{am}}-{T_w}} \right)} \right]} \over Q} + c}$$

with ${\displaystyle{{d{T_w}} \over {dt}}}$ corresponding to the evolution over time of the day maximum WT (*T*_w_). Time here is a period of +7 days and −7 days around an extreme date identified using GAM modeling (*i.e*., a period of 15 days). *T*_am_ = day minimum air temperature, *T*_aM_ = day maximum air temperature, *Q* = streamflow. *a*, *b* and *c* are model variables representing respectively day thermal sensitivity, night thermal sensitivity, and non-convective thermal flux (Georges et al., 2021).

The model includes three composite variables. Variable *a* relates to the day convective heat flux, which corresponds to a heat transfer from a hot to a cold body depending on the temperature difference. Variable *a* modulates the temperature difference between the day maximum air and water temperature (*i.e*., daytime convective flux). Variable *b* also relates to the convective heat flux at night, when the air temperature typically leads to a lowering of WT. In what follows, variables *a* and *b* will be respectively denoted ‘day thermal sensitivity’ and ‘night thermal sensitivity’. Thermal sensitivity is the response of WT to changes in air temperature (Kelleher et al., 2012; Chang & Psaris, 2013). Variable *c* (denoted ‘non-convective thermal flux’) was added to the equation to incorporate other physical processes influencing WT such as advection not specifically studied because of scant data availability. The model was fitted to extreme events covering the different seasons (37) identified by Georges et al. (2021) at each measurement site (92) during a 6-year period to estimate specific best fit of variables *a, b* and *c*. WT data collected by a dense network recording WT at pre-determined intervals of 10 minutes between 2012 and 2018 was used (Fig. 1).

**Figure 4:**
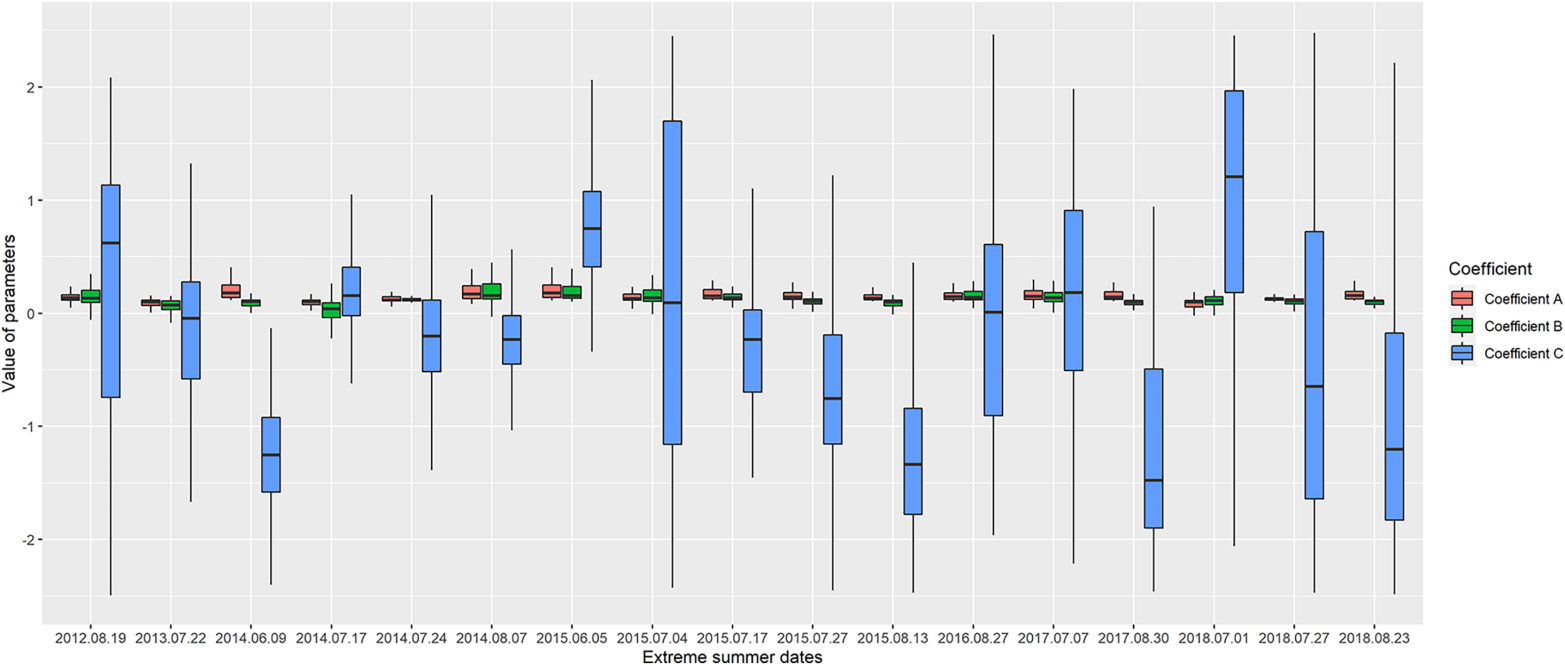
Boxplot of the three parameters variability in the measurement stations for each extreme date.

In this study, a total of 1,564 values (17 extreme summer events (Table 1) × 92 measurement sites) of the three variables were studied in relation with environmental variables in order to improve our understanding of the temporal thermal dynamics around extreme events. Figure 4 shows the range of these model variables in the study area between the different summer extreme events.

### Dataset: environmental variables studied

A set of environmental variables characterizing land cover, topography (channel slope, elevation), hydromorphology (channel sinuosity, water level, watershed area, baseflow index) and shade conditions, identified in previous studies as the most impactful drivers influencing WT were considered as potentially able to explain the model variables. The water flow and air temperature variables used to build the differential equation (Georges et al., 2021) were therefore not retained as explanatory variables. The explanatory variables taken are spatially and temporally distinct. Spatially, they fall into three groups: area-dependent, station scale-dependent, and stream network-dependent.

#### Area-dependent variables

Land cover was evaluated for the entire upstream catchment area and then characterized for the different spatial scales studied. Land cover classes of each watershed were obtained from the Lifewatch project (version 2.10, 2015) (Delangre, Radoux & Dufrêne, 2017; Radoux & Bogaert, 2017). To facilitate analysis and interpretation, the 18 land cover classes were aggregated into eight categories: herbaceous, broadleaved, needle-leaved, agricultural land, water and flooded land, artificialized land, mixed forest, and other (Table 2). The proportion of each land cover category was then computed within the watershed of each measurement site for each spatial scale.

**Table 1:** Description of the selected variables including variability in space and time. “Time dependent” indicates if the variable varies over time (X = yes). “Variation within spatial scales” indicates if the variable varies spatially among *CA (the entire upstream catchment), ripa (50 m wide corridors on each sides of the stream along the entire upstream river network), buff_1 (CA + circular buffer of 1 km), buff_1_ripa (ripa + the circular buffer of 1 km), buff_2* (CA + circular buffer of 2 km) and *buff_2_ripa (ripa + the circular buffer of 2 km)* scales (X = yes) (Fig. 5).

Variables	Description (units)	Time dependent	Variation between spatial scales
**SHADE**	Mean shade on the stream (°)	X	X^1^
**LEVEL**	Water level at the measurement site (m)	X	
**SINUO**	Sinuosity of the watercourse		X^1^
**Landcover**			
**AGRI**	Proportion of crop and culture cover (%)		X
**ARTI**	Proportion of artificial land cover (%)		X
**BROAD**	Proportion of broadleaved forests (%)		X
**HERBA**	Proportion of herbaceous land cover (%)		X
**FORESTS**	Proportion of mixed forests (%)		X
**NEEDLE**	Proportion of needleleaved forests (%)		X
**OTHER**	Proportion of mixture vegetation, bare land, recently cleared areas with forest regrowth and Xmas trees (%)		X
**WATER**	Proportion of water and flooded land (%)		X
**AREA**	Watershed area (km²)		
**SLOP**	Local channel slope (%)		
**ELEV**	Topographic elevation at the measurement site (m)		
**BFI**	Base flow index		

**Note:**

^1^Variable between spatial scales except between CA and ripa, between buff_2 and buff_2_ripa and between buff_1 and buff_1_ripa.

**Table 2:** Dates of the 17 summer extreme events analyzed in this study among the 37 extreme events covering the different seasons identified by Georges et al. (2021).

Seasons/extreme events	Summer
	2012-08-19
	2013-07-22
	2014-06-09
	2014-07-17
	2014-07-24
	2014-08-07
	2015-06-05
	2015-07-04
	2015-07-17
	2015-07-27
	2015-08-13
	2016-08-27
	2017-07-07
	2017-08-30
	2018-07-01
	2018-07-27
	2018-08-23
**Total**	**17 events**

#### Station scale-dependent variables

The watershed area of each measurement site was calculated using the sf package (Pebesma & Bivand, 2018) in the R environment. The study area was also characterized by the local channel slope and the elevation at the measurement site scale using QGis (QGIS Development Team, 2020). Topographic metrics were obtained using the digital terrain model (2014, grid resolution: 1 m × 1 m). For slope, the ratio of the difference in elevation between two points (downstream and upstream of the WT site measurement) to the channel length (about 4 km) was calculated. Water level was obtained from the WT measurement sites measuring water level every 10 min along with WT and aggregated daily by the median. The baseflow index (BFI) was introduced as a hydrological indicator corresponding to the ratio of the low flow to the total river flow (Beaufort et al., 2020). As used by Beaufort et al. (2020), BFI was calculated in our study to reflect the influence of groundwater. The BFI lies between 0 and 1. A low index corresponds to a watershed where storage is low and where the watershed is closely governed by climatic events (Beaufort et al., 2020). Conversely, watersheds with a high BFI contain storage areas, aquifers and reservoirs. These underground elements mitigate the effects of flow change. BFI was calculated for each measurement station according to the definition of Gustard, Bullock & Dixon (1992) using the R package *lfstat* version 0.9.4 (Koffler, Gauster & Laaha, 2016) and the function *BFI*.

#### Stream network-dependent variables

Stream network-dependent variables characterize the whole watercourse of the river and fluctuate correspondingly. Stream sinuosity and shade belong to this category.

We extracted the stream sinuosity, namely the ratio of the length of the river to the length of the mean axis of the watercourse (Malavoi & Bravard, 2011). Sinuosity was calculated according to this definition in a previous study by Michez et al. (2017) in the study area for navigable and non-navigable watercourses (drainage area > 1 km²) (Fig. 1). In order to determine a mean stream sinuosity value to characterize the stream network upstream from each measurement site, the sinuosity values were weighted by each of the upstream reach lengths.

To quantify shade from riparian vegetation, valley morphology and channel orientation, the ‘analytical hillshading’ method from SAGA (Tarini, Cignoni & Montani, 2006) was used. Based on a digital elevation model (photogrammetric digital surface model derived from regional orthophoto survey from the year 2016 processed as described in Michez et al. (2020), grid resolution: 5 m × 5 m) and sun position, the tool calculates angles at which sunlight impinges on the surface. The values range between 0 and 90°, with 90° corresponding to an area in shadow. A shade map was created for each extreme date at solar noon. The different shade maps were then masked following the water surface extent of the stream network in the drainage area of the measurement site. The water surface was mapped by Michez et al. (2017) for river reaches with a drainage area above 50 km² threshold based on an airborne LiDAR point cloud. For smaller rivers (drainage area > 1 km² and < 50 km²), the water surface was mapped based on a fixed buffer (2 m) applied over the river network lines. The median shade value was computed within the watershed of each measurement site for each extreme date and spatial scale.

Temporally, these environmental variables can be considered either constant or variable over time. Constant variables were assumed to be unchanging over the time period studied (2012–2018). They comprised all the variables except shade and water level. These time-dependent variables were calculated only for the days of interest (*i.e*., the extreme events).

### Spatial scales of environmental variables

**Figure 5:**
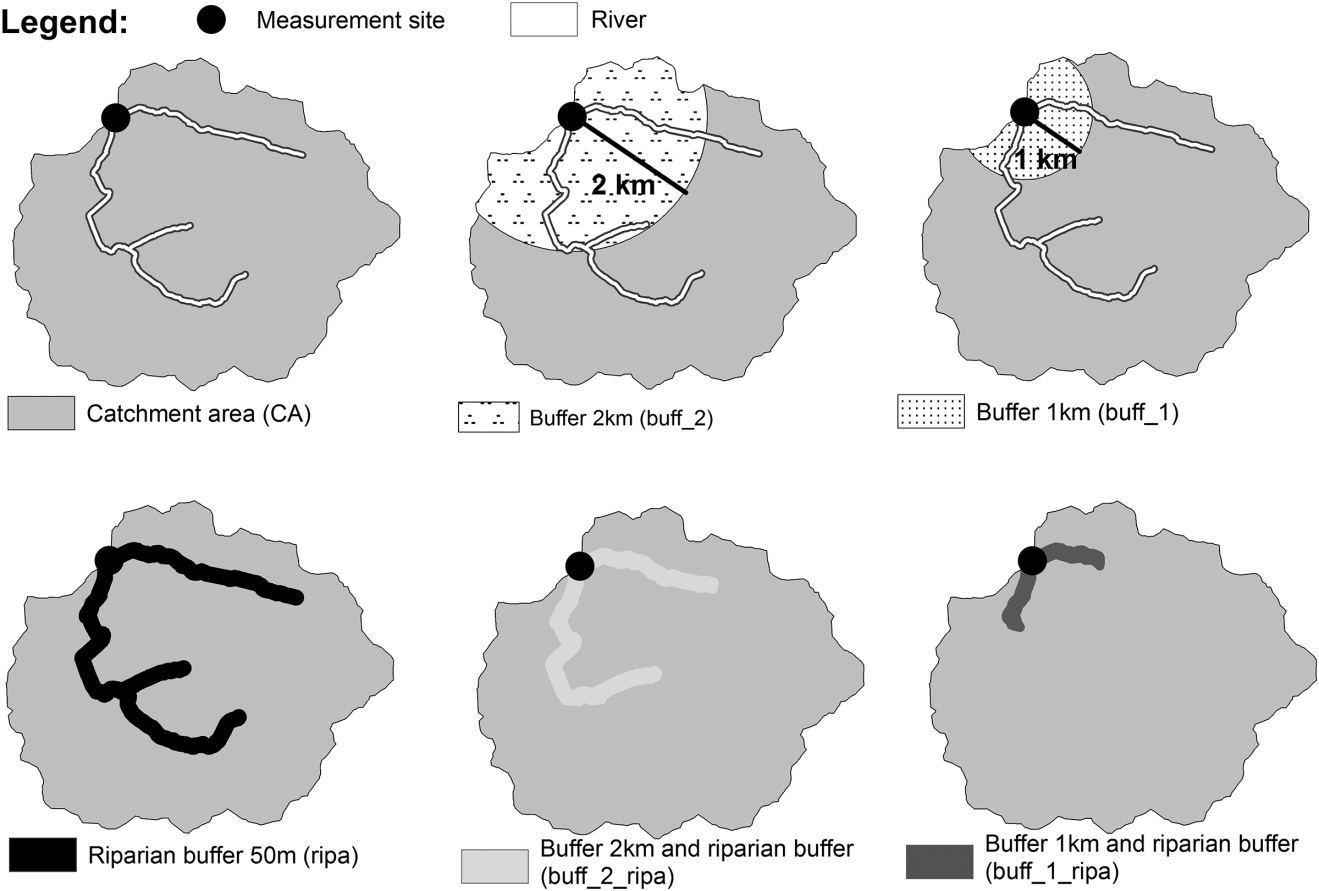
Example of a watershed showing one measurement site and the different spatial scales: catchment area (*CA*), buffer zones with different width (*buff_1* and *buff_2*), riparian buffer of 50 m on each side of the watercourse (*ripa*) and the combined riparian buffer with 1 km and 2 km buffer (*buff_1_ripa and buff_2_ripa*).

We then investigated the role of environmental variables at different spatial scales (Fig. 5) to determine whether the local environment had a greater influence on the WT thermal dynamic than the upper reaches or the whole catchment. Five area-dependent variables were measured only at the site measurement scale: watershed area, local channel slope, elevation, water level and baseflow index. All the others were extracted from six spatial scales:

- Catchment scales: the entire upstream catchment (***CA***) and “circular” buffer of 1 km (***buff_1***) and 2 km (***buff_2***). The circular buffer corresponds only to the part intersecting the watershed, upstream of the WT site measurement;
- Riparian buffer scales: 50 m wide corridors on each side of the stream along the entire upstream river network (***ripa***), within the circular buffer of 1 km (***buff_1_ripa***), and 2 km (***buff_2_ripa***) upstream river.

The spatial scales were designated based on previous studies (Wu et al., 2020; Chang & Psaris, 2013; Sliva & Williams, 2001; Scott et al., 2002), context of rivers studied, and field investigation.

The different environmental variables were calculated at the different spatial scales using R (R Core Team, 2018) and QGIS (QGIS Development Team, 2020).

Because some variables (riparian shade and water level) are time-dependent as stated above, and different spatial scales are studied, Table 2 summarizes the variables and their potential variation in space and time. Like the model variables, the environmental variables were also calculated for each measurement site and at each extreme event when the variable depended on it.

### Statistical analysis

Results were examined in terms of seasonal timescales focusing on summer when the solar radiation is strongest; the vegetation is fully grown (maximum shade) and the ecological impact most critical for the aquatic ecosystem (Langan et al., 2001; Meisner, 1990; Miara et al., 2018). The extreme dates highlighted by Georges et al. (2021) were therefore assigned to a meteorological season (winter: December–February, spring: March–May, summer: June–August, autumn: September–November). For the summer period (Table 1), a single value of each environmental variable was retained for each measurement site (all extreme dates combined). Only shade and water level varied temporally. They were therefore aggregated by the mean to obtain a single value for each measurement site. Finally, 1,656 observations were listed (92 measurement sites × 18 variables (15 environmental variables + 3 model variables)).

Relationships between model variables (day thermal sensitivity, night thermal sensitivity, and non-convective thermal flux) and environmental variables were evaluated for the six spatial scales during summer with stepwise multiple linear regressions (MLR) to ensure non-collinearity. Prior to all statistical analysis, environmental data were standardized to zero mean and unit variance.

To understand the relationship between environmental variables and the model variables, stepwise MLRs were carried out for each combination of model variable and spatial scale (24 models: six spatial scales × three variables). The effectiveness of these 24 models was assessed based on the adjusted *R*-squared value (Adj. *R*²). In the stepwise selection, best influential environmental variables based on stepwise AIC values were selected. The significance of each variable selected from the stepwise procedure was assessed by MLR with the associated *p*-value. The statistical significance was conventionally set at 0.05.

The estimate value of each variable retained by the stepwise MLR was analyzed. Estimate value corresponds to a coefficient quantifying the influence of the variable taking into account the influence of all the others.

Statistical analyses were performed using R version 3.5 (R Core Team, 2018).

A file with the model variables (variables to be explained) and the environmental variables (explanatory variables) is provided for each spatial scale as a supplemental file. The data is standardized as explained in the methodology. The description of the different column (variables) is to be considered with Table 2.

## Results

### Descriptive analysis

The watersheds studied in the analysis covered a wide range of environmental conditions. The result of descriptive statistical analysis is presented in Fig. 2. Figure 3 shows the different land cover classes of the watersheds studied.

Most stations were located in watersheds ranging between 56 ha and 115,207 ha and at low elevation (<200 m). Water levels measured were low in summer (<0.5 m) but could reach 1.06 m. On the other hand, most of the watersheds had a high BFI, the values being closer to 1 than 0. Watersheds were therefore mainly composed of aquifers with groundwater storage. Sinuosity ranged around 1.15 (moderately meandering), but covered a wide range of conditions from straight to strongly meandering (Horacio, 2014).

Land cover of the 92 watersheds studied showed a broad diversity. Northern watersheds were mainly composed of cropland, while forest and grassland dominated in the south and east.

### Influence of environmental variables and spatial scale on the thermal sensitivity variables

To understand the influence of environmental variables and spatial scales on the three model variables, stepwise MLRs were conducted. Results of the stepwise MLR between model and environmental variables are shown in Table 3.

**Table 3:** Relations between environmental variables and parameters a, b or c during summer for different spatial scales through MLR.

Spatial scale	SHADE	SINUO	AGRI	ARTI	BROAD	HERBA	FORESTS	NEEDLE	OTHER	WATER	AREA	SLOP	LEVEL	ELEV	BFI	Adj. R² (%)
	Parameter a, daily thermal sensitivity															
CA	−0.18*	0.17*	0.27^#^	0.37**	0.51**	0.78*			0.64**		0.61***		0.19*			61.4
Buff_2	−0.19*		0.15	0.19^#^	0.21	0.37*			0.43**	−0.11	0.56***		0.28**			59.5
Buff_1	−0.166*								0.183**		0.526***		0.303***			57.8
Ripa	−0.24**		1.35*	1.43*	1.53*	1.09*	0.25^#^	0.95^#^	0.99**		0.61***		0.24**			59.3
Buff_2_ripa	−0.16*								0.13^#^		0.58***		0.24**			56.7
Buff_1_ripa	−0.13^#^										0.56***		0.27**	0.13^#^		56.1
	Parameter *b*, night thermal sensitivity															
CA			0.29	0.59**	0.43^#^	1.25**			0.95**		0.18^#^			0.39*	−0.38***	26.3
Buff_2			1.44^#^	1.32*	1.61*	2.61*		1.62*	0.91**						−0.39***	24.2
Buff_1	−0.16	−0.29**		−0.14					0.21^#^			−0.16		0.19	−0.14	23.0
Ripa	−0.16		1.36^#^	1.62*	1.41^#^	1.33^#^	0.33	0.01	1.35**						−0.31**	20.4
Buff_2_ripa			0.64^#^	0.67^#^	0.58	0.26		0.61**	0.42*			−0.17			−0.25*	16.2
Buff_1_ripa		−0.28**						0.17				−0.21*		0.28*	−0.17^#^	20.3
	Parameter *c*, non-convective thermal flux															
CA		0.25*	−0.18^#^						0.25*		−0.19^#^					13.2
Buff_2			−0.16						0.42***		−0.14					16.1
Buff_1					−0.17^#^		0.24*		0.43***		−0.14					22.9
Ripa				0.19		0.29^#^	0.24^#^		0.69***		−0.16					18.7
Buff_2_ripa								0.39***								14.3
Buff_1_ripa			−0.35*	−0.48**	−0.43**	−0.19^#^										8.82

**Note:**

The grey boxes correspond to the variables selected by the stepwise analysis based on the AIC. Statistical significance, based on *p*-value, is symbolized by stars (“***”: *p*-value [0, 0.001]; “**”: *p*-value [0.001, 0.01]; “*”: *p*-value [0.01, 0.05]; “^#^”: *p*-value [0.05, 0.1]). The value specified is the estimate of the corresponding linear coefficient.

#### Model variable *a*, day thermal sensitivity

The three most significant environmental variables influencing the day thermal sensitivity for all the spatial scales were watershed area (AREA), water level (LEVEL) and shade (SHADE) (Table 3). AREA and LEVEL had a positive correlation, while SHADE influenced the day thermal sensitivity negatively.

The proportion of mixed vegetation, bare land, recently cleared areas with forest regrowth and Xmas trees (OTHER) also significantly influenced day thermal sensitivity except for two spatial scales (Buff_2_ripa and Buff_1_ripa). The OTHER estimate was positive for the retained spatial scales (Table 3).

For the other variables, the trends were less clear. Some of them were selected as a result of the stepwise process but their significance was very low (‘#’ in Table 3).

Adjusted *R*-squared (Adj. *R*²) values ranged between 55% and 62%. The highest Adj. *R*² value concerned the model at the spatial scale of the watershed (CA). However, the difference in explanation from the model with the lowest Adj. *R*² (buff_1_ripa, Table 3) was not sufficient to dissociate the results according to the spatial scale.

#### Model variable *b*, night thermal sensitivity

Unlike day thermal sensitivity, night thermal sensitivity was significantly influenced by the baseflow index (BFI) at the different spatial scales studied (Table 3). The correlation was always negative. OTHER also significantly controlled the night thermal sensitivity with a positive correlation. Other variables had less influence on night thermal sensitivity.

Adj. *R*² values were lower than those obtained for the day thermal sensitivity MLR but, as above, there was not enough difference between the Adj. *R*² of the models to highlight a spatial scale.

#### Model variable *c*, non-convective thermal flux

The adjusted *R*-squared (Adj. *R*²) value was low, usually less than 20%. Environmental variables at the 1 km buffer scale (buff_1) explained the non-convective thermal flux slightly more (Table 3).

The results of stepwise MLR failed to establish clear trends. Few environmental variables had a significant influence at any spatial scale.

However, the OTHER land cover class had a significant influence at almost all spatial scales. OTHER was statistically positively correlated with the non-convective thermal flux.

## Discussion

Our results demonstrate that the environmental variables studied mainly influenced day and night thermal sensitivities. For the non-convective thermal flux, the result suggests that the added variable *c* was not specific enough to be explained by the environmental variables studied. Our detailed discussion will therefore focus on day and night thermal sensitivities *a* and *b*.

### Influence of other variables

Our results show that at most 60% of the temporal thermal dynamic variability was explained by the environmental variables studied (Table 3). This result suggests that environmental variables other than those studied also influenced thermal dynamics around extreme events. For example, thermal sensitivity is also influenced by human activities and thermal pollution (from industries), deforestation, flow modification and global change. Abstraction of water from a river reduces water volume and so thermal capacity. These modifications lead to an increased maximum WT and a decreased minimum WT (Dymond, 1984). Dymond (1984) analyzed the WT changes associated with abstraction of water by modeling the downstream WT from upstream data (upstream WT, net energy flux, discharge) and reusing this model, varying the discharge to quantify the change in WT due to water abstraction.

Prediction of WT under reduced flow, due for example to irrigation, hydroelectric power stations, or water withdrawal, was also analyzed by Bartholow (1991), Morin, Nzakimuena & Sochanski (1994), and Hockey, Owens & Tapper (1982).

Water releases (reservoirs, dams, industrial effluent) have also been shown to influence thermal downstream conditions (Lowney, 2000).

### Day thermal sensitivity *a*

Watershed area (AREA) was a very highly significant variable influencing day thermal sensitivity at all spatial scales (Table 3). Hilderbrand, Kashiwagi & Prochaska (2014) found no relationship between thermal sensitivity and basin size. Nevertheless, the increase in day thermal sensitivity with the size of the watersheds is explained by stream size. Larger watersheds have gentler hillslopes and larger streams, with therefore less forest shading (Chang & Psaris, 2013; Culler et al., 2018). In addition, thermal sensitivity increased with AREA because the time spent in the river was longer, implying a longer equilibration of WT with air temperature (Beaufort et al., 2020). Time spent determines the amount of exposure to radiation (Wehrly, Wiley & Seelbach, 2006).

Shade controls day thermal sensitivity during summer. Studied with the other environmental variables, the correlation between shade and day thermal sensitivity was negative. A correlation analysis showed that only forest land cover was significantly correlated with shade (correlation = 0.41; *p* = 0). The other variables had no influence on shade.

Most prior research studied the influence of shade on maximum stream WT (Caissie, 2006; Rutherford et al., 2004; Li et al., 1994; Abell & Allan, 2002; Hrachowitz et al., 2010). However, few studies have addressed the influence of riparian shade on thermal sensitivity as here (Chang & Psaris, 2013; Beaufort et al., 2020). Beaufort et al. (2020) also demonstrated that the thermal sensitivity of a stream decreased with the riparian vegetation, which captures solar radiation.

Wawrzyniak et al. (2017) modeled the shade cast by the vegetation on the river surface using LiDAR data to study its thermal effect. Their results show that one tenth of solar radiation was intercepted by riparian vegetation. This corresponds to a temperature decrease of 0.26 ± 0.12 °C (summer 2010) and 0.31 ± 0.18 °C (summer 2011). Riparian shade retains direct solar inputs and heating (Hilderbrand, Kashiwagi & Prochaska, 2014), so the watercourse heats up less quickly. Net radiation can be five times lower in a stream under shading riparian forest than in an unshaded stream (Moore, Spittlehouse & Story, 2005). Our study confirms that thermal sensitivity is controlled by shade mainly during summer because solar heating (solar radiation) is more intense then (Needham & Jones, 1959; Beschta, 1997; Wawrzyniak et al., 2017).

Our first hypothesis is thus supported by our stepwise MLR showing the influence of different environmental variables on day thermal sensitivity *a*.

### Night thermal sensitivity *b*

The proportion of storage area, aquifers and reservoirs compared to watersheds with low storage represented by the BFI index in Table 3 influenced night thermal sensitivity negatively. Decreased BFI is associated with increased night thermal sensitivity, meaning that watersheds with small storage areas are more sensitive. In other studies, Kelleher et al. (2012), Chang & Psaris (2013), and Beaufort et al. (2020) analyzed BFI as a measure of groundwater contribution, in relation to thermal sensitivity. They also demonstrated that thermal sensitivity increased with decreasing groundwater contribution.

Other studies specifically analyzed the relationship between groundwater inflows and thermal sensitivity (Luce et al., 2014). Erickson & Stefan (2000) demonstrated that groundwater inflows limited the influence of air temperature on WT, lowering thermal sensitivity. In their study on the thermal sensitivity of Pennsylvania streams, Kelleher et al. (2012) showed that narrow annual variation (about 3 °C) was observed in some sites with high groundwater inputs. These observations can be explained by the fact that groundwater inflow supplies water at constant temperature, which exerts a stabilizing influence on stream WT (Erickson & Stefan, 2000). WT change in response to air temperature change is reduced, causing a decrease in thermal sensitivity and an increase in thermal inertia (O’Driscoll & DeWalle, 2004).

In summer, hyporheic exchanges supply groundwater that is generally cooler than mainstream water (Mayer, 2012). Thermal and water exchanges occur in the stream’s internal structure because the aquifer is hydraulically connected to the main channel (Boano et al., 2006). A back-and-forth transfer therefore occurs between the stream and the aquifer (Gibert, Danielopol & Stanford, 1994; Cristea & Janisch, 2007). Other studies have found that hydraulic properties of the aquifer are a driver of thermal sensitivity in streams (Kelleher et al., 2012; Mayer, 2012; Cristea & Janisch, 2007). The total amount of groundwater contained in aquifers depends on rainfall, the intensity of extraction (agriculture, factories, etc.), and on the nature of the subsoil (SPW Agriculture, Ressources naturelles et Environnement, 2020). For example, in a region with a permeable subsoil, such as chalk, the proportion of precipitation reaching the groundwater is very high, as opposed to a schist region (SPW Agriculture, Ressources naturelles et Environnement, 2020). Johnson (2004) found that maximum WT in the upstream bedrock reach was up to 8.6 °C higher than maximum WT in the downstream alluvial reach and 3.4 °C lower for minimum WT (distance between measurement stations: 350 m). However, sedimentation of the channel, for example through forest harvesting, can decrease the influence of hyporheic exchange through stream bed clogging (Moore & Wondzell, 2005).

Although significant control of BFI is observed for night thermal sensitivity, groundwater inflows also influence day thermal sensitivity. The studies cited above refer to day WT (Kelleher et al., 2012; Chang & Psaris, 2013; Beaufort et al., 2020). No significant effect of the BFI on day thermal sensitivity was observed in our study, probably because our watersheds were large enough to allow greater stream interaction with the surrounding environment (especially shade) during the day (Erickson & Stefan, 2000), masking the influence of daytime groundwater inflows.

Our first hypothesis is thus supported by our stepwise MLR showing the influence of different environmental variables on night thermal sensitivity.

### Management insights

The analysis of model variables reflecting thermal sensitivity makes it possible to highlight the most influential environmental variables. The variables studied are not likely to be all equally important in the different watersheds and spatial scales. Determining which environmental variables most influence WT and at which spatial scales is necessary to provide management advice for counteracting heating due to climate change.

Shade, watershed area, water level and BFI were identified as the most influential variables. Among these variables, shade and water level (and more generally the river morphology) are the only relevant variables on which a manager can easily act. O’Briain, Shephard & Coghlan (2017) found that shallower sites showed greater WT extremes, together with sites with little riparian tree cover. Our results are consistent with those of O’Briain, Shephard & Coghlan (2017), suggesting that the thermal resilience of rivers can be improved by riparian vegetation (shade) and restored channel morphology.

Typical stream morphology restoration focuses on reinstating geomorphic features (riffle/pool, meanders) and modifying width-to-depth ratio (Malavoi & Bravard, 2011). O’Briain, Shephard & Coghlan (2017) showed that restoration of riparian tree cover is a more sustainable approach because it allows natural recruitment of large wood debris, recognized as a catalyst and a restoration tool impacting channel morphology within a few years (Osei, Harvey & Gurnell, 2015). Riparian tree cover also provides shade, mitigating WT rise. Moreover, acting on riparian cover is commonly recognized as an accessible management tool in terms of application, cost and time for environment managers (O’Briain, Shephard & Coghlan, 2017).

In restored wood-laden rivers (large wood debris), Osei, Harvey & Gurnell (2015) have demonstrated that the channel does not remain wide. Fine sediment accumulates in and around wood jams and flow velocities increase, leading to the mobilization of fine sediment. Pool formation and sediment bar deposition also occur in wood-restored streams (Elosegi et al., 2017; Pilotto et al., 2016).

Besides wood debris production, riparian vegetation produces shade. Shade cast by trees reduces energy inputs to the river and so counteracts thermal extremes. This observation was also made in other studies (Broadmeadow et al., 2011; Hannah et al., 2008; Garner et al., 2015). Future river management needs to preserve (Feld et al., 2018) and include riparian planting (Brown et al., 2010; Garner et al., 2015; Johnson & Wilby, 2015). For example, Feld et al. (2018) review the use of fences to avoid riparian degradation by livestock. Romasco-Kelly (2018) reports in the Walla Walla basin of southeastern Washington (USA) that local organizations set up riparian restoration projects to use shade from trees to diminish thermal pollution.

Merely advocating the plantation and upkeep of riparian vegetation oversimplifies the actions to be implemented by managers. Using vegetation as a tool to reduce heat energy input into the water requires considering different ‘vegetation variables’ influencing the shade provided (Wondzell, Diabat & Haggerty, 2019). Rutherford, Meleason & Davies-Colley (2018) show the influence of the ratio of tree height to stream width on stream shade. The shade effect is more pronounced on narrow streams (<5 m wide) (Whitledge et al., 2006). According to Feld et al. (2018), length, width, and density should also be considered, as should species (LeBlanc, Brown & FitzGibbon, 1997; Lenane, 2012). Collins et al. (2006) made a literature search on riparian functional width, and showed that the average recommended or observed maximum riparian width for the water body cool function was 40 m. The literature review of Sweeney & Newbold (2014) concludes that buffers ≥30 m wide are needed for full protection against thermal change. Chang & Psaris (2013) found that riparian shading was only significantly negatively correlated with thermal sensitivity 1 km upstream relative contributing area scale.

The effect of riparian vegetation on WT can also vary depending on external factors: stream flow, channel morphology, anthropogenic action (grazing, harvest), and hydraulic structures (reservoirs, dams) (Justice et al., 2017; Wondzell, Diabat & Haggerty, 2019). The orientation of the watercourse (included in our shade calculation) will also influence the amount of shade on the river. As demonstrated by Pluhowski (1970), a north-south watercourse receives less sunshine than an east-west. In the Northern Hemisphere, south bank vegetation maximizes shade on the stream (Larson & Larson, 1996). Streams moderated by groundwater inflows are less sensitive to riparian shade (Johnson & Wilby, 2015).

Besides mitigating thermal pollution, acting on riparian vegetation improves the connectivity between rivers and their floodplains (Opperman et al., 2010), and reduces, retains (Booth & Leavitt, 1999), and filters urban runoff (Welsch, 1991).

While the riparian zone influences thermal sensitivity, among other things, specific management features must be considered to make strategic choices (Beaufort et al., 2020). It is not necessary to maintain or plant a riparian buffer around all streams. Managers need to plan their investments (finance and staffing workforce) strategically. Adding vegetation will be ineffective against thermal heating if other factors are more impactful, such as groundwater flows or stream configuration (width, orientation, topographical conditions, etc.) (Johnson & Wilby, 2015). However, trees can be planted as a priority where there is no vegetation and where the BFI is low (no storage).

### Spatial scale

Concerning the spatial scale, Sliva & Williams (2001) demonstrate that variables at the catchment scale have slightly greater influence on water quality than those at the buffer scale (100 m). Pratt & Chang (2012) show that the watershed scale has a greater effect on water quality than the buffer scale (100 m riparian) except for WT. In their review on relationships between land use, spatial scale and stream macroinvertebrate communities, Sponseller, Benfield & Valett (2001) found that land cover influenced mean and maximum WT within respectively riparian corridors and riparian subcorridors (200 m).

The studies cited above show the difficulty defining the most influential spatial scale for management. Although choosing the spatial scale is controversial, as observed by Chang & Psaris (2013), our study shows that managers can focus on relatively small geographical areas.

Although no one spatial scale appears clearly more influential than any other in our analyses (Adj. *R*² in Table 3), the near stream area appears sufficient to impact thermal sensitivity. The environment across the entire riparian or the entire watershed spatial scales does not significantly explain temporal thermal dynamics around extreme events any better than the local scale, meaning that large scale management will not be more efficient. Because the financial and time/labor costs need to be considered in a management project, the results of our study suggest that managers should focus on small spatial scales. However, the too-small number of environmental variables (three: landcover, sinuosity and shade) at different spatial scales cannot validate our second hypothesis.

## Conclusion

This work was motivated by the need to understand the environmental variables that influence temporal thermal dynamics during extreme events and so respond to future increases in air temperature.

Even though the influence of environmental variables on river thermal heterogeneity has been extensively studied, temporal WT dynamics during extreme events and at different spatial scales is still not fully understood.

This study investigates the relationship between a set of environmental variables: land cover, topography (slope, elevation), hydromorphology (sinuosity, water level, watershed area, BFI), and shade and thermal dynamic models of Georges et al. (2021) applied on 92 measurement sites in Wallonia (southern Belgium) and 17 summer extreme dates between 2012 and 2018.

Shade, BFI and watershed area were the most significant variables influencing thermal sensitivity. Since managers can only act on a few environmental variables, the findings of our study can be used to help make natural, cost-effective decisions on ways to reduce thermal extremes.

To reduce thermal sensitivity, restoring and preserving the vegetation cover, which limits solar radiation on the watercourse, is a cost-effective solution, although the effect of shade depends on the riparian forest (species, height, density) and the morphological properties (orientation, width) of the river. Management at a small spatial scale (50 m riparian buffer) should be strategically promoted (finance and staffing) because a larger management scale is not more effective in reducing thermal sensitivity to extreme events at specific points.

## Supplemental Information

10.7717/peerj.12494/supp-1 Supplemental Information 1 Dataset 1: Values of parameters of the model (3) and environmental variables (15) at the 1 km buffer spatial scale.

10.7717/peerj.12494/supp-2 Supplemental Information 2 Dataset 2: Values of parameters of the model (3) and environmental variables (15) at the 2 km buffer spatial scale.

10.7717/peerj.12494/supp-3 Supplemental Information 3 Dataset 3: Values of parameters of the model (3) and environmental variables (15) at the watershed (CA) spatial scale.

10.7717/peerj.12494/supp-4 Supplemental Information 4 Dataset 4: Values of parameters of the model (3) and environmental variables (15) at the riparian scale (50 m) included in the 1 km buffer spatial scale.

10.7717/peerj.12494/supp-5 Supplemental Information 5 Dataset 5: Values of parameters of the model (3) and environmental variables (15) at the riparian scale (50 m) included in the 2 km buffer spatial scale.

10.7717/peerj.12494/supp-6 Supplemental Information 6 Dataset 6: Values of parameters of the model (3) and environmental variables (15) at the riparian scale (50 m).

## Additional Information and Declarations

### Data Availability

The following information was supplied regarding data availability: The raw data is available in the Supplemental Files.
